# Mutation of Gly195 of the ChlH Subunit of Mg-chelatase Reduces Chlorophyll and Further Disrupts PS II Assembly in a Ycf48-Deficient Strain of *Synechocystis* sp. PCC 6803

**DOI:** 10.3389/fpls.2016.01060

**Published:** 2016-07-20

**Authors:** Tim S. Crawford, Julian J. Eaton-Rye, Tina C. Summerfield

**Affiliations:** ^1^Department of Biochemistry, University of OtagoDunedin, New Zealand; ^2^Department of Botany, University of OtagoDunedin, New Zealand

**Keywords:** ChlH, Ycf48, chlorophyll biosynthesis, PS II, *Synechocystis*

## Abstract

Biogenesis of the photosystems in oxygenic phototrophs requires co-translational insertion of chlorophyll *a*. The first committed step of chlorophyll *a* biosynthesis is the insertion of a Mg^2+^ ion into the tetrapyrrole intermediate protoporphyrin IX, catalyzed by Mg-chelatase. We have identified a *Synechocystis* sp. PCC 6803 strain with a spontaneous mutation in *chlH* that results in a Gly195 to Glu substitution in a conserved region of the catalytic subunit of Mg-chelatase. Mutant strains containing the ChlH Gly195 to Glu mutation were generated using a two-step protocol that introduced the *chlH* gene into a putative neutral site in the chromosome prior to deletion of the native gene. The Gly195 to Glu mutation resulted in strains with decreased chlorophyll *a*. Deletion of the PS II assembly factor Ycf48 in a strain carrying the ChlH Gly195 to Glu mutation did not grow photoautotrophically. In addition, the ChlH-G195E:ΔYcf48 strain showed impaired PS II activity and decreased assembly of PS II centers in comparison to a ΔYcf48 strain. We suggest decreased chlorophyll in the ChlH-G195E mutant provides a background to screen for the role of assembly factors that are not essential under optimal growth conditions.

## Introduction

In oxygenic photosynthesis Photosystem II (PS II) and Photosystem I (PS I) catalyze the conversion of light energy into the chemical energy that is required for carbon fixation. The reaction center (RC) and core antenna proteins of both PS II and PS I require the co-translational insertion of chlorophyll *a* for correct folding (Eichacker et al., 1996; Sobotka, 2014; Yang et al., 2015). In addition, the co-ordinated production of chlorophyll and chlorophyll-binding proteins is essential to prevent the accumulation of potentially detrimental unbound chlorophyll and chlorophyll precursors. Although a large proportion of newly synthesized chlorophyll *a* is associated with PS I, the assembly of PS II and chlorophyll biosynthesis are synchronized (He and Vermaas, 1998). There are 70 chlorophyll *a* molecules in the PS II pigment-protein complex along with at least 20 protein subunits and additional cofactors (Umena et al., 2011; Shen, 2015). Assembly of PS II involves the formation of four pre-complexes, each containing one of the four chlorophyll *a*-binding proteins that are found in the mature complex: D1 and D2 that form the RC, and CP43 and CP47 that function as a core antenna (Boehm et al., 2011). The pre-complexes are assembled, beginning with the association of the D1- and D2-containing modules, to form larger assembly intermediates and finally the mature photosystem (Komenda et al., 2012). In addition, the D1 subunit is the site of light-induced photo-oxidative damage and a repair cycle enables the removal and replacement of damaged D1 to maintain PS II activity (Komenda et al., 2012). Part of this repair cycle involves the removal of chlorophyll *a* from damaged D1, as well as insertion of chlorophyll *a* into new D1 subunits (Yao et al., 2012).

The chlorophyll *a* biosynthesis pathway is comprised of at least 15 enzymatic steps, many of which are shared with the biosynthetic pathway for heme (Sobotka, 2014; Fujita et al., 2015). The first dedicated step in the synthesis of chlorophyll *a* is the insertion of Mg^2+^ into protoporphyrin IX (PP IX) to form Mg-PP IX; this reaction is catalyzed by Mg-chelatase (Jensen et al., 1996). This represents a branch point that contributes to the regulation of tetrapyrrole biosynthesis by controlling the partitioning of precursor molecules such as PP IX into different biosynthetic pathways (Shepherd et al., 2005). Mg-chelatase is a large protein complex consisting of three different subunits CHLI, CHLD, and CHLH. Both CHLI and CHLD form hexamers that associate with a single catalytic CHLH subunit (Wang and Grimm, 2015). The corresponding ChlH protein in cyanobacteria is comprised of an N-terminal ‘head’ domain and a larger domain that forms a hollow, cage-like structure that binds porphyrin and a similar domain organization is expected in plant CHLH (Qian et al., 2012; Chen et al., 2015). Regulation of the Mg-chelatase enzyme occurs through association of the CHLH subunit with the GUN4 protein, in an interaction that stimulates enzyme activity (Larkin et al., 2003; Sobotka et al., 2008). In addition, in plants, CHLH is involved in plastid-nuclear signaling and in cyanobacteria ChlH has a regulatory role as an anti-sigma factor, binding to the RNA polymerase sigma factor SigE in the light (Mochizuki et al., 2001; Osanai et al., 2009). Two mutant lines of *Arabidopsis thaliana* with mutations in *CHLH* have been described: the Pro to Leu mutation in *cch* plants corresponds to the Pro595 residue in ChlH from the cyanobacterium *Synechocystis* sp. PCC 6803 (hereafter *Synechocystis* 6803) and the Ala to Val substitution in the *gun5* mutant corresponds to the Ala942 residue. Both Pro595 and Ala942 are located in the central cage-like structure of the protein and may interfere with the chelation reaction due to spatial hindrance (Chen et al., 2015). In *A. thaliana* these mutations caused reductions in chlorophyll levels of ∼70 and ∼30% in the *cch* and *gun5* mutants, respectively (Mochizuki et al., 2001).

Re-sequencing of *Synechocystis* 6803 has shown genetic differences have arisen between wild-type sub-strains under laboratory conditions (Jones, 2014). This occurrence of spontaneous mutations has produced several mutations in a sub-strain, GT-O2, including in the *chlH* gene (Morris et al., 2014). The mutation in *chlH* results in a Gly195 to Glu amino acid substitution in a conserved region of the ChlH protein. The GT-O2 strain was able to grow photoautotrophically, although at a slightly slower rate and reaching a lower OD_730 nm_ compared to its parental strain, GT-O1 (Morris et al., 2014). In this report, we show the construction and characterization of *Synechocystis* 6803 strains carrying this ChlH Gly195 to Glu mutation. In addition, we investigated the impact of impairing biogenesis of PS II in the ChlH mutant by deleting the assembly factor Ycf48. We selected the Ycf48 protein [HCF136 in *A. thaliana* and the first assembly factor discovered (Meurer et al., 1998)] because it is transiently associated with the D1 pre-complex during assembly and repair of PS II. This factor binds to the D1 precursor polypeptide (pD1) and stabilizes formation of the RC II complex containing the D1 and D2 pre-complex modules (Komenda et al., 2008; Mabbitt et al., 2014). We hypothesized that deletion of Ycf48 in a strain where chlorophyll *a* supply is reduced may impact on both PS II biogenesis and the PS II repair cycle.

## Materials and Methods

### *Synechocystis* 6803 Growth Conditions and Construction of Mutant Strains

Strains of *Synechocystis* 6803 were maintained on BG-11 medium agar plates supplemented with 5 mM glucose, 20 μM atrazine, 10 mM TES-NaOH (pH 8.2), 0.3% sodium thiosulfate and appropriate antibiotics. Liquid cultures were grown photoautotrophically or photomixotrophically (with 5 mM glucose) in BG-11 liquid medium with appropriate antibiotics, as previously described (Eaton-Rye, 2011; Summerfield et al., 2013).

Generation of *chlH* mutants was performed in two steps. Amplicons were generated using overlap-extension PCR (Bryskin and Matsumura, 2010), in which most of the *slr0168* gene was deleted and replaced by a spectinomycin-resistance cassette (**Supplementary Figures S1A,B**). The *slr0168* gene site has been used as a neutral site for integration of genes into the chromosome previously with no reported effect on phenotype (Kunert et al., 2000). A HindIII restriction site was introduced into the 5′ end of the spectinomycin-resistance cassette using PCR. This amplicon was ligated into pGEM-T Easy and the resulting *Δslr0168*:specR plasmid was transformed into wild-type *Synechocystis* 6803 (GT-O1) to create the Δ*slr0168* neutral site control strain (**Supplementary Figure S1B**). The *slr1055* (*chlH*) gene was amplified from the GT-O1 and GT-O2 wild types (Morris et al., 2014), using primers designed to introduce HindIII restriction sites at the 5′ and 3′ ends of the amplicons (**Supplementary Table S1**). These amplicons and the Δ*slr0168*:specR plasmid, were digested with HindIII and ligated to produce the ChlH-G195G:specR and ChlH-G195E:specR plasmids, respectively. The resultant plasmids were transformed into *Synechocystis* 6803 strains (GT-O1 or GT-O2) to introduce either the unmodified *chlH* (in the GT-O1:G195G and GT-O2:E195G strains) or the mutant *chlH* gene (in the GT-O1:G195E and GT-O2:E195E strains) into the putative neutral site (**Supplementary Figure S1**). In the second step, a Δ*chlH* plasmid was constructed using overlap-extension PCR in which a chloramphenicol-resistance cassette was located between the sequences up- and downstream of the native *chlH* gene (**Supplementary Figures S1C,D**). This plasmid was transformed into the strains which contained a copy of the *chlH* gene in the neutral site to produce strains which each contained only one copy of *chlH*, either the GT-O1 (Gly195) or GT-O2 (Gly195 to Glu) variant. A summary of the mutants created in this study is presented in **Table 1.**

**Table 1:** Strains of *Synechocystis* sp. PCC 6803 used in this study.

Strain	Background	Insertion at neutral site (*slr0168*)	Gene at native *chlH* site (*slr1055*)	Reference
GT-O1	*-*	*-*	*chlH* (G195)	Morris et al., 2014


GT-O2	*-*	*-*	*chlH* (E195)	Morris et al., 2014


Δ*slr0168*	GT-O1	spec^R^	*chlH* (G195)	This study


GT-O1:G195G	GT-O1	*chlH* (G195) + spec^R^	cam^R^ (Δ*chlH*)	This study


GT-O1:G195E	GT-O1	*chlH* (E195) + spec^R^	cam^R^ (Δ*chlH*)	This study


GT-O2:E195G	GT-O2	*chlH* (G195) + spec^R^	cam^R^ (Δ*chlH*)	This study


GT-O2:E195E	GT-O2	*chlH* (E195) + spec^R^	cam^R^ (Δ*chlH*)	This study




*spec^R^ and cam^R^ refer to spectinomycin-resistance and chloramphenicol-resistance cassettes, respectively.*

Construction of the Δ*ycf48* (Δ*slr2034*) plasmid has been described previously (Jackson et al., 2014); this plasmid was transformed into the GT-O1:G195G and GT-O1:G195E strains to produce the GT-O1:G195G:ΔYcf48 and GT-O1:G195E:ΔYcf48 strains, respectively. Colony PCR and sequencing were used to confirm the mutant genotypes as appropriate (data not shown).

### Physiological Measurements

Photoautotrophic growth, whole-cell absorption spectra, oxygen evolution, and chlorophyll *a* fluorescence analyses were performed as previously described (Crawford et al., 2016). Cells grown photomixotrophically to mid-exponential phase were washed and re-suspended to an optical density at 730 nm (OD_730 nm_) of 1.5 in BG-11 supplemented with 25 mM HEPES-NaOH (pH 7.5) for physiological measurements. Chlorophyll *a* was quantified by extraction in methanol and measurement at 663 nm (MacKinney, 1941). Low-temperature (77 K) fluorescence emission spectroscopy was performed using sodium fluorescein as an internal standard as previously described (Crawford et al., 2016). Traces were normalized to the fluorescein emission maxima at 505 nm.

### Protein Analyses

For analysis of PS II assembly, thylakoid membranes were isolated from photomixotrophically grown cultures, solubilized with β-dodecylmaltoside and separated by blue-native polyacrylamide gel electrophoresis (BN-PAGE) as previously described (Crawford et al., 2016). Proteins were transferred to polyvinylidene fluoride (PVDF) membranes for 1 h at 25 V in the presence of 0.1% SDS, and were subjected to immunodetection using antibodies to PS II and PS I core protein subunits (Jackson and Eaton-Rye, 2015).

## Results

### A Strain with an Altered Whole-Cell Spectrum Had Accumulated Several Mutations Including a *chlH* Mutation Changing Residue Gly195 to Glu

**FIGURE 1:**
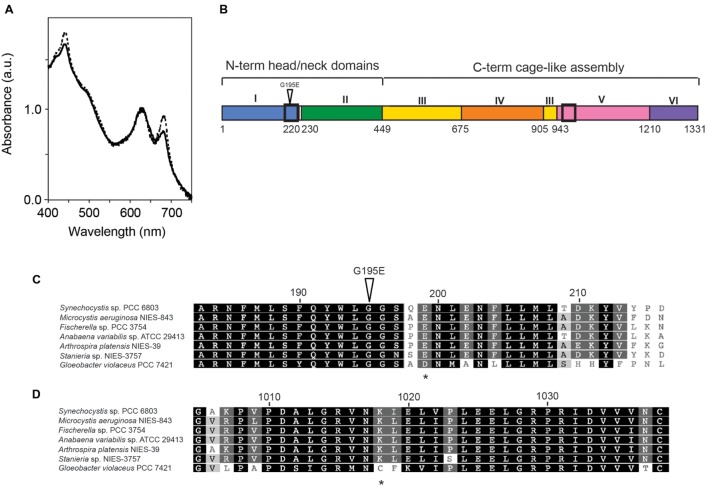
**The *Synechocystis* sp. PCC 6803 GT-O2 strain has an altered whole-cell absorption spectrum and contains a *chlH* mutation resulting in a Gly195 to Glu amino acid substitution.** **(A)** Whole-cell absorption spectra of the GT-O1 (dotted line) and GT-O2 cells (solid line) grown photoautotrophically to mid-exponential phase in 40 μE.m*^-^*^2^.s*^-^*^1^ constant white light. Spectra representative of three independent biological replicates are shown normalized to the phycobilin absorption maxima at 625 nm. **(B)** Schematic representation showing the domains of the ChlH subunit of the Mg-chelatase enzyme from *Synechocystis* sp. PCC 6803. Black boxes indicate the positions of the sections expanded in **(C,D)**, and the position of the Gly195-Glu mutation is indicated by an inverted triangle (Chen et al., 2015). **(C,D)** Amino acid sequence alignment of sections of domain I **(C)** or domain V **(D)** of cyanobacterial ChlH proteins. Amino acids are numbered according to the ChlH protein of *Synechocystis* sp. PCC 6803. The position of the Gly195 to Glu mutation present in the GT-O2 strain is indicated by an inverted triangle, and the positions of the Glu199 and Lys1018 residues, which form the inter-monomer salt bridge contact, are indicated by asterisks.

Comparison of two strains in our laboratories, GT-O1 and GT-O2, showed differences in whole-cell absorption spectra. Both strains exhibited absorption maxima at 625 nm, as well as at 435 and 685 nm, corresponding to phycobilins and chlorophyll *a*, respectively (**Figure 1A**). The GT-O2 strain exhibited a reduction in the peaks at 435 nm and 685 nm corresponding to chlorophyll *a* absorbance but not the 625 nm phycobilin peak (**Figure 1A**). Six unique genome sequence variants are present in the GT-O2 strain compared to GT-O1 cells (Morris et al., 2014). These are predicted to result in amino acid changes in five proteins: the long-chain fatty acid CoA ligase, FadD (Slr1609, Arg641 to Gln); the serine metalloprotease, HtrA/DegP (Slr1204, frameshift after amino acid 66 of 452); hypothetical protein Slr0154 (Ser307 to Phe); the histidine kinase, Hik8 (Sll0750, Arg65 to Cys), and the Mg-chelatase catalytic subunit, ChlH (Slr1055, Gly195 to Glu). The Mg-chelatase enzyme catalyzes the first committed step in chlorophyll *a* biosynthesis, therefore the ChlH Gly195 to Glu mutation was a candidate for the decreased absorbance of chlorophyll *a* in the GT-O2 wild type.

The ChlH Gly195 residue is located in the α6 helix of the N-terminal ‘head’ domain I (**Figure 1B**; **Supplementary Figure S2**). This Gly195 residue was conserved in ChlH proteins representing all five sections of cyanobacteria, and is part of a conserved protein region (**Figure 1C**). Although chiefly monomeric in solution, the crystal structure of ChlH obtained by Chen et al. (2015) indicated that this protein can form a dimer, and that interactions occur exclusively between the N-terminal domain I and the domain V of opposing monomers (**Supplementary Figure S2**). Dimerization of ChlH subunits is mediated by polar interactions, including six hydrogen bonds and a salt bridge between the Glu199 and Lys1018 residues of opposing monomers. The Gly195 residue is in close proximity to Glu199 (**Supplementary Figure S2**); the Glu199 residue is conserved in many cyanobacteria, but is substituted for an Asp in the cyanobacterium *Gloeobacter violaceus* PCC 7421. The Lys1018 residue was found in the aligned cyanobacterial ChlH protein sequences except that of *G. violaceus* PCC 7421 (**Figure 1D**).

### Generation of Mutations in *chlH* with a Two-Step Process Involving Insertion into the Putative Neutral Site at *slr0168*

**FIGURE 2:**
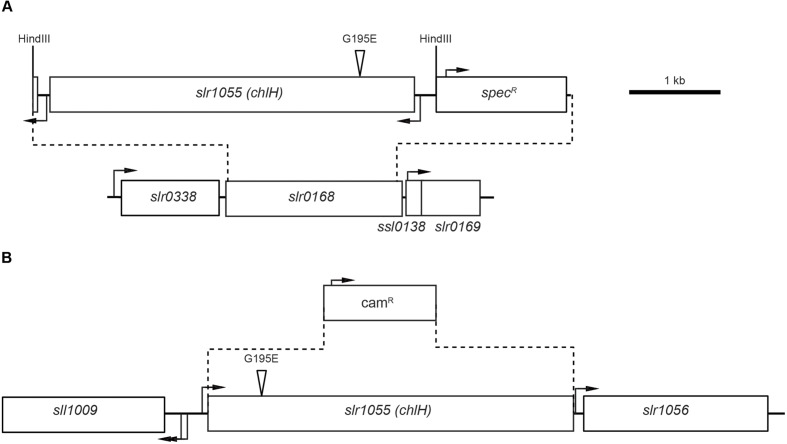
**Construction of the *chlH* mutant strains of *Synechocystis* sp. PCC 6803.** **(A)** Genomic structure of the *chlH* insertion into the *slr0168* neutral site region of the chromosome. Dotted lines indicate the replacement of *slr0168* with a sequence containing either the unmodified *chlH* gene or the copy encoding the Gly195 to Glu substitution (amplified from the GT-O1 or GT-O2 strains, respectively) and a spectinomycin-resistance cassette. The boxes represent predicted ORFs and transcriptional start sites, based on data published in Kopf et al. (2014), are indicated by black arrows. The position of the Gly195 to Glu substitution is indicated by a triangle, and the introduced HindIII site in the spectinomycin-resistance cassette into which the amplified *chlH* genes were ligated is indicated. **(B)** Genomic structure of the *slr1055* (*chlH*) region in the *chlH* mutant strains. Dotted lines indicate the deletion of the *chlH* ORF and its replacement with a chloramphenicol-resistance cassette. For a more detailed explanation of this process see Supplementary Information and **Supplementary Figure S1**.

To determine the effect of the ChlH Gly195 to Glu mutation *in vivo*, a copy of either the unmodified gene (encoding Gly195) or mutant *chlH* gene (encoding the Gly195 to Glu mutation) was introduced at a putative neutral site (*slr0168*) in the *Synechocystis* 6803 genome (Kunert et al., 2000). A spectinomycin-resistance cassette inserted downstream of the *chlH* gene enabled selection (**Figure 2A**). Once the introduced *chlH* gene had been fully segregated, the native gene was deleted and a chloramphenicol-resistance cassette was inserted in place of this copy of *chlH* (**Figure 2B**). This method was used to generate strains containing only the GT-O1 copy of *chlH*, encoding Gly195, in both the GT-O1 and GT-O2 backgrounds. Similarly, strains were generated containing only the GT-O2 copy of *chlH*, encoding the Gly195 to Glu substitution, in both the GT-O1 or GT-O2 backgrounds. Mutants in the GT-O2 background enabled us to determine the impact of restoring the Gly195 to this background, as well as, including an additional control strain where the Glu195 was introduced into ChlH in the neutral site. The sequences of the oligonucleotides used for the construction and verification of these strains are in Table S1. In addition, a Δ*slr0168* control strain was produced which contained only the spectinomycin-resistance cassette in the neutral site and which retained the native *chlH* gene at its wild-type locus.

### A Mutation That Changes ChlH Residue 195 from Gly to Glu Altered the Whole-Cell Spectrum

**FIGURE 3:**
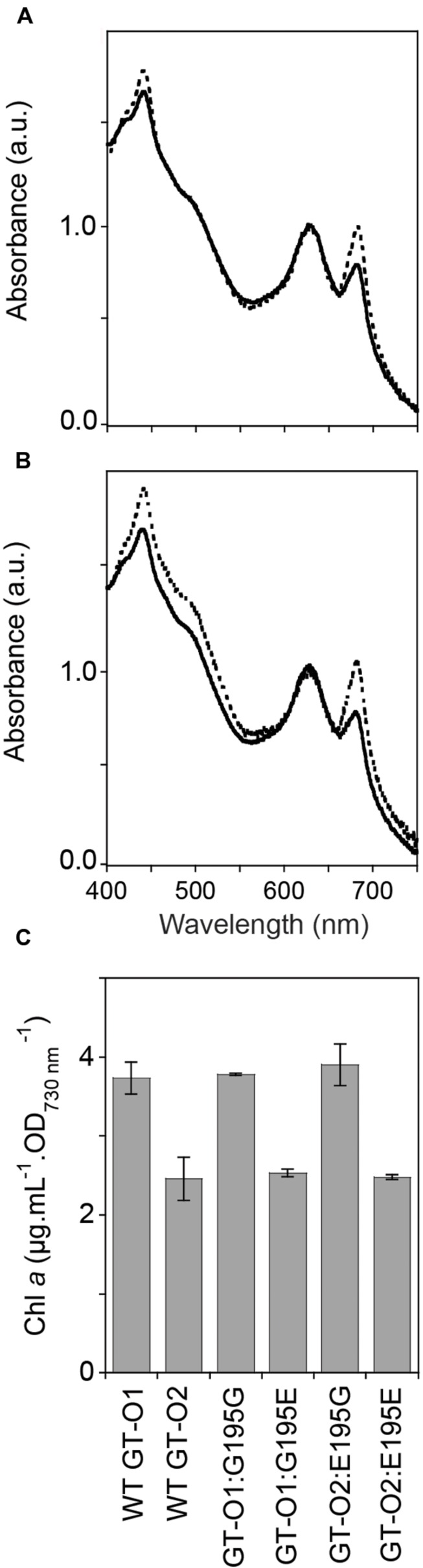
**Pigment composition of the *chlH* mutant strains of *Synechocystis* sp. PCC 6803.** Cells were grown photoautotrophically to mid-exponential phase in BG-11 (25 mM HEPES-NaOH, pH 7.5). **(A)** Whole-cell absorption spectra of the GT-O1:G195G (dotted line) and GT-O1:G195E strains (solid line). **(B)** Whole-cell absorption spectra of the GT-O2:E195G (dotted line) and GT-O2:E195E strains (solid line). Spectra representative of three independent biological replicates are shown normalized to the phycobilin absorption maxima at 625 nm. **(C)** Levels of chlorophyll *a* in photoautotrophically grown cells, determined by measuring the absorbance of methanol-extracted chlorophyll at 663 nm. The data shown are the mean [Chl *a*] per optical density of 1.0 at 730 nm ± the standard error of the mean (SEM) from three independent biological replicates.

**FIGURE 4:**
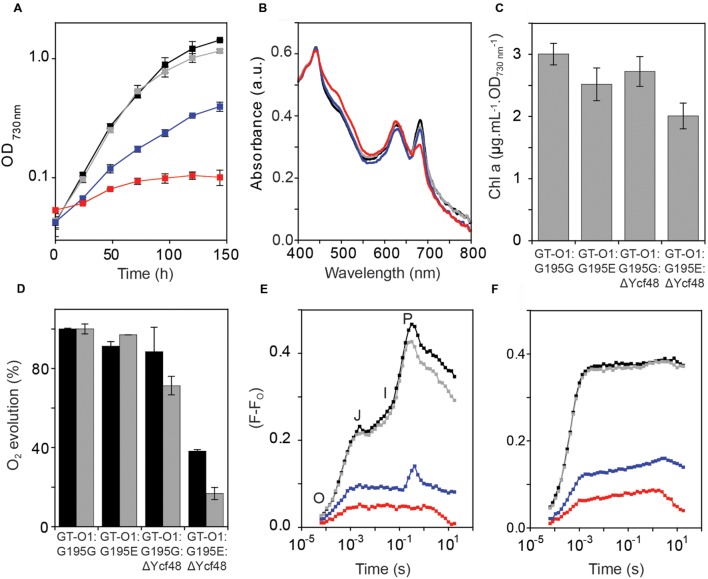
**Phenotype of the ChlH-ΔYcf48 mutant strains of *Synechocystis* sp. PCC 6803.** The strains were: GT-O1:G195G: black; GT-O1:G195E: gray; GT-O1:G195G:ΔYcf48: blue; GT-O1:G195E:ΔYcf48: red. **(A)** Photoautotrophic growth of the ChlH mutants in unbuffered BG-11 liquid media. Cultures were incubated in constant white light at an intensity of 40 μE.m*^-^*^2^.s*^-^*^1^ at 30^o^C, and their optical density at 730 nm was measured every 24 h. The data are the mean OD_730 nm_ ± the SEM. **(B)** Whole-cell absorption spectra of photomixotrophically grown strains. Representative spectra are shown, normalized to the absorption maxima at 435 nm. **(C)** Levels of chlorophyll *a* in photomixotrophically-grown cells, determined by measuring the absorbance of methanol-extracted chlorophyll at 663 nm. The data shown are the mean [Chl *a*] per optical density of 1.0 at 730 nm ± the SEM. **(D)** Oxygen evolution of strains was measured in the presence of either 15 mM NaHCO_3_ (black) or 200 μM DMBQ and 1 mM K_3_Fe(CN)_6_ (gray) as electron acceptors. Data shown are the mean rate of oxygen evolution over the 1st minute of actinic illumination ± the SEM. Rates are shown as a percentage of that measured in the GT-O1:G195G strain (278 and 293 μmol O_2_.mg Chl*^-^*^1^.h*^-^*^1^ in the presence of bicarbonate and DMBQ, respectively). **(E,F)** Room temperature chlorophyll *a* fluorescence induction of strains, measured with a 455 nm measuring pulse in the absence **(E)** or presence **(F)** of 40 μM DCMU. The O (origin), J, I (inflections), and P (peak) features of the fluorescence transient are indicated. Representative traces are shown normalized to the same initial fluorescence level (F_O_).

The whole-cell spectra of the GT-O1 wild type and Δ*slr0168* control strain were similar (data not shown). Introduction of unmodified *chlH*, under its own promoter, to produce the GT-O1:G195G strain using the *slr0168* site resulted in a whole-cell spectrum similar to that observed with GT-O1 cells, whereas introduction of the ChlH Gly195 to Glu mutation to produce the GT-O1:G195E strain resulted in decreased chlorophyll *a* absorption at both the 435 and 685 nm maxima compared to the GT-O1 strain (**Figure 3A**). Conversely, the introduction of unmodified *chlH* into the GT-O2 background resulted in the GT-O2:E195G strain which had increased chlorophyll *a* maxima that were similar to the GT-O1 wild type (cf. **Figures 1A** and **3B**). In addition, the GT-O2:E195G strain showed an increased absorption in the 475–520 nm range compared to the GT-O2:E195E strain suggesting elevated carotenoid levels. Furthermore, the spectrum of the GT-O2:E195E strain was similar to spectra from the GT-O2 and GT-O1:G195E strains, suggesting that the presence of the Gly195 to Glu variant in ChlH results in a decrease in chlorophyll *a* absorbance (**Figures 1A** and **3A,B**). The quantification of methanol-extracted chlorophyll *a* showed that the Gly195 to Glu mutation in ChlH resulted in a ∼35–40% reduction of chlorophyll *a* accumulation in the GT-O2 and the GT-O1:G195E and GT-O2:E195E strains, in comparison to the GT-O1 and the GT-O1:G195G and GT-O2:E195G strains in samples normalized to an OD_730 nm_ of 1.0 (**Figure 3C**). The Gly195 to Glu mutation had little effect on the rate of photoautotrophic growth of the GT-O1:G195E strain, although these cells reached a slightly lower final OD_730 nm_ in stationary phase (**Figure 4A**); this is consistent with that observed in the GT-O2 wild type (Morris et al., 2014). The photoautotrophic growth of the Δ*slr0168* control strain was indistinguishable from the GT-O1 wild type and the GT-O1:G195G strain (data not shown). Using flow cytometry and electron microscopy we did not detect any difference in size between strains containing Gly195 or Glu195 (data not shown), therefore the OD_730 nm_ should represent a similar cell number in all strains compared.

### Introduction of the ChlH Gly195 to Glu Mutation Abolished Photoautotrophic Growth in a ΔYcf48 Strain

Formation of mature PS II is a multistep process involving the co-ordinated assembly of several intermediate complexes that require the insertion of chlorophyll *a*. The Ycf48 assembly factor is associated with the RC pre-complex; however, the absence of this factor does not completely block PS II assembly (Komenda et al., 2008; Jackson et al., 2014; Sobotka, 2014). We determined whether deletion of Ycf48 in a strain with reduced chlorophyll *a* would impact on PS II biogenesis and the PS II repair cycle. Deletion of Ycf48 resulted in reduced photoautotrophic growth, with a decrease in the final OD_730 nm_ reached in stationary phase from 1.43 ± 0.02 in the GT-O1:G195G background to 0.40 ± 0.04 for the GT-O1:G195G:ΔYcf48 strain (**Figure 4A**); this is consistent with other ΔYcf48 strains characterized (Komenda et al., 2008; Jackson et al., 2014; Jackson and Eaton-Rye, 2015). However, deletion of Ycf48 from the GT-O1:G195E mutant resulted in a strain that reached an OD_730 nm_ of only 0.1 ± 0.02 under photoautotrophic growth conditions, indicating increased sensitivity of cells to the absence of this PS II assembly factor when the ChlH Gly195 to Glu mutation is present.

Whole-cell absorption spectra of photomixotrophically grown cells showed a slight decrease in the 685 nm absorption of chlorophyll *a* in the GT-O1:G195E strain relative to the GT-O1:G195G strain, when normalized to the absorbance at 435 nm (**Figure 4B**). The GT-O1:G195G:ΔYcf48 mutant had a whole-cell absorption spectrum with decreased absorption maxima at 625 and 685 nm compared to the GTO1:G195G strain. The GT-O1:G195E:ΔYcf48 strain’s whole-cell spectrum was the most altered, exhibiting a decreased 685 nm maxima and an increased carotenoid shoulder compared to the GT-O1:G195G:ΔYcf48 strain (**Figure 4B**). Levels of chlorophyll *a* measured for each strain at the same OD_730 nm_ showed a 16% decrease in the GT-O1:G195E strain relative to the GT-O1:G195G strain (**Figure 4C**). Deletion of Ycf48 resulted in a small decrease in chlorophyll *a* levels in the GT-O1:G195G:ΔYcf48 strain, whereas in a GT-O1:G195E:ΔYcf48 strain there was a ∼26% decrease in chlorophyll *a* compared to the GT-O1:G195G strain.

### Oxygen Evolution and Variable Chlorophyll *a* Fluorescence Induction Were Reduced in the GT-O1:G195E:ΔYcf48 Mutant

Oxygen evolution was measured in mixotrophically grown cultures. The rates of oxygen evolution were similar in the GT-O1:G195E and GT-O1:G195G strains, when measured in the presence of 15 mM bicarbonate (measuring whole-chain electron transport) or 200 μM of the artificial quinone 2,5-dimethyl-1,4-benzoquinone (DMBQ) (measuring PS II-specific electron transport) (**Figure 4D**). In the GT-O1:G195G:ΔYcf48 strain, oxygen evolution was decreased, to 89 ± 12 and 71 ± 5% of the GT-O1:G195G strain in the presence of bicarbonate and DMBQ, respectively. Removal of Ycf48 in a ChlH-G195E background resulted in rates of oxygen evolution that were reduced, to 38 ± 1 and 17 ± 3% of the rates for the GT-O1:G195G strain in the presence of bicarbonate and DMBQ, respectively.

The activity of the acceptor side of PS II was analyzed by measuring variable chlorophyll *a* fluorescence induction. Fluorescence induction transients of the GT-O1:G195G and GT-O1:G195E strains differed by only a slightly decreased P level in the latter (**Figure 4E**). It was previously reported that removal of Ycf48 resulted in a considerable decrease in the variable fluorescence level and this was observed in the GT-O1:G195G:ΔYcf48 strain (Jackson et al., 2014). The variable fluorescence induction of the GT-O1:G195E:ΔYcf48 strain was further reduced compared to the GT-O1:G195G:ΔYcf48 strain and did not display an I-P rise (**Figure 4E**). In the presence of 40 μM 3-(3,4-dichlorophenyl)-1,1-dimethylurea (DCMU), which blocks electron transfer beyond Q_A_ of PS II, variable fluorescence was similar in the GT-O1:G195G and GT-O1:G195E strains, reduced in the GT-O1:G195G:Ycf48 strain and further decreased in the GT-O1:G195E:ΔYcf48 strain (**Figure 4F**). Variable fluorescence in the presence of DCMU provides an indication of the numbers of assembled PS II centers and these data are consistent with decreased centers in both strains lacking Ycf48, with the lowest number of centers in the GT-O1:G195E:ΔYcf48 strain.

### The 77 K Fluorescence Emission Spectra Were Altered in the GT-O1:G195E:ΔYcf48 Mutant

**FIGURE 5:**
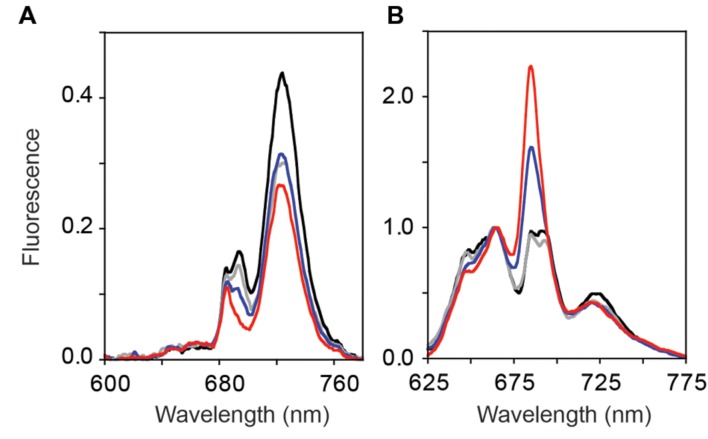
**Low-temperature (77 K) fluorescence emission spectra of the ChlH mutant strains of *Synechocystis* sp. PCC 6803.** Photomixotrophically grown cells of the GT-O1:G195G (black), GT-O1:G195E (gray), GT-O1:G195G:ΔYcf48 (blue) and GT-O1:G195E:ΔYcf48 strains (red) were excited with either a 440 nm **(A)** or 580 nm excitation light **(B)**. Representative spectra are shown normalized to either the 505 nm emission maximum of a 250 nM sodium fluorescein internal standard **(A)** or the 665 nm phycocyanin emission **(B)**.

Reduced PS II-specific oxygen evolution and variable fluorescence in the GT-O1:G195G:ΔYcf48 and the GT-O1:G195E:ΔYcf48 strains are consistent with decreased numbers of assembled PS II centers in these strains. Low-temperature (77 K) fluorescence emission spectroscopy was performed on photomixotrophically grown cultures using 250 nM sodium fluorescein as an internal standard. Introduction of the ChlH Gly195 to Glu mutation had little effect on the 685 and 695 nm fluorescence emission in the GT-O1:G195E strain with a 440 nm excitation light (**Figure 5A**). The 685 nm emission peak is attributed to unassembled pre-complexes and the CP43 core antenna protein, while the emission at 695 nm arises from the CP47 core antenna and is indicative of fully assembled PS II RCs (Boehm et al., 2011; D’Haene et al., 2015). The fluorescence emission maximum observed at 725 nm originates from PS I, and this was decreased by ∼35% in the GT-O1:G195E strain compared with the GT-O1:G195G strain when spectra were normalized to the emission maximum of fluorescein at 505 nm (**Figure 5A**). A similar decrease in the emission at 725 nm was observed in the GT-O1:G195G:ΔYcf48 strain and this strain also displayed a decreased emission at 685 nm with a further decrease at 695 nm. This is consistent with impaired assembly and/or repair of PS II in the absence of Ycf48; however, the decrease in fluorescence emission from PS I suggests an additional effect on PS I assembly due to the removal of this assembly factor. Deletion of Ycf48 in a ChlH-G195E background resulted in a complete absence of the 695 nm emission in the GT-O1:G195E:ΔYcf48 strain, suggesting a decreased number of PS II centers; this strain also showed a decreased PS I emission maxima at 725 nm, compared to the GT-O1:G195G:ΔYcf48 strain.

Steady-state phycobilisome-coupled energy transfer was assessed by low-temperature emission spectra following excitation at 580 nm. When normalized to the fluorescence emission maximum at 665 nm, little difference was observed between the emission spectra of the GT-O1:G195G and GT-O1:G195E strains, except for a slight decrease in the 725 nm emission originating from PS I in the latter, consistent with the decrease in PS I emissions observed in 440 nm-excited cells (**Figure 5B**). In the GT-O1:G195G:ΔYcf48 strain, an increase in the fluorescence emission at 685 nm was observed; this emission originates from the ApcE terminal emitter of the phycobilisome antenna and indicates an increased level of uncoupled phycobilisomes in this strain (Shen et al., 1993; Jackson et al., 2014). This emission was further increased in the GT-O1:G195E:ΔYcf48 strain, and both this and the GT-O1:G195G:ΔYcf48 strains displayed the decreased PS I emission seen in the GT-O1:G195E strain.

### BN-PAGE Confirmed PS II and PS I Assembly was Altered in the GT-O1:G195E:ΔYcf48 Mutant

**FIGURE 6:**
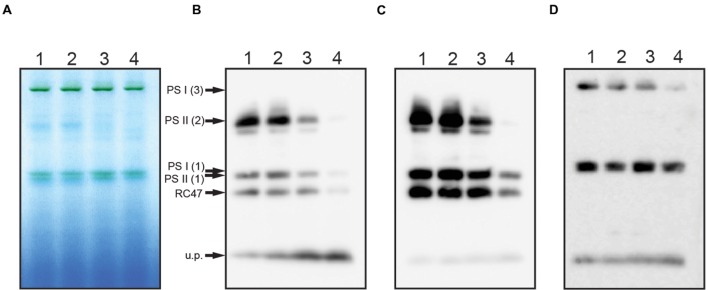
**Assembly of PS II and PS I complexes in the ChlH mutant strains of *Synechocystis* sp. PCC 6803.** Thylakoid membranes, isolated from photomixotrophically grown cultures, were solubilized with 1.0% β-D-dodecylmaltoside and 25 μg protein equivalent separated on 3–12% NativePAGE gel **(A)**. Proteins were transferred to PVDF membranes and subjected to immunodetection using antibodies to the D1 **(B)** or D2 subunits of PS II **(C)**, or the PsaA subunit of PS I **(D)**. Lanes: 1: GT-O1:G195G; 2: GT-O1:G195E; 3: GT-O1:G195G:ΔYcf48; 4: GT-O1:G195E:ΔYcf48. Putative photosystem multimers and assembly intermediates are indicated by arrows: PS I (3): trimeric PS I; PS II (2): dimeric PS II; PS I (1) and PS II (1): monomeric PS I and PS II, respectively; RC47: CP43-less PS II repair/assembly intermediate; u.p., Unassembled protein.

To further analyze the PS II complexes in the ChlH mutant strains, thylakoid membranes were analyzed using BN-PAGE and western blotting. In the GT-O1:G195G and GT-O1:G195E strains, the D1 and D2 core subunits were detected at similar levels in dimeric and monomeric PS II complexes, and in a CP43-less assembly/repair intermediate, RC47 (**Figures 6B,C**). In the GT-O1:G195G:ΔYcf48 strain the D1 and D2 proteins detected in PS II dimers and monomers were reduced, and the unassembled D1 slightly increased. However, only low levels of PS II monomers and only trace amounts of dimers were detected in the GT-O1:G195E:ΔYcf48 strain, consistent with the very low levels of PS II activity, while a low level of the RC47 complex was present. In addition, an antibody to the PsaA subunit of PS I revealed that the ratio of PS I monomers to trimers in the GT-O1:G195G:ΔYcf48 appeared to increase and that trimers were greatly reduced or destabilized in the GT-O1:G195E:ΔYcf48 mutant (**Figure 6D**).

## Discussion

### A Strain with the ChlH Gly195 to Glu Mutation has Less Chlorophyll *a*

The ChlH subunit of Mg-chelatase catalyzes the first committed step in chlorophyll *a* biosynthesis and is involved in the regulation of gene expression in cyanobacteria (Jensen et al., 1996; Osanai et al., 2009). Commensurate with the importance of ChlH, segregation of gene-targeted knockouts has not been successful (Kong and Xu, 2002). Instead we have used a two-step approach to construct mutant strains with an inactivated *chlH* locus but which contain a *chlH* gene, expressed from a neutral site and encoding either an unmodified ChlH protein or the Gly195 to Glu variant. Characterization of these mutants revealed that introduction of the Gly195 to Glu mutation decreased the accumulation of chlorophyll *a*, with a more pronounced decrease in photoautotrophically grown cells compared to mixotrophically grown cultures. Our approach is readily applicable to the *in vivo* study of other targeted mutants in *chlH* from *Synechocystis* 6803.

The ChlH-Pro595 to Leu variant (equivalent to the substitution in the *A. thaliana cch* mutant) and the ChlH-Ala942 to Val change (equivalent to the substitution in the *A. thaliana gun5* mutant) have both been studied in the *Synechocystis* ChlH subunit *in vitro* (Davison and Hunter, 2011). In the *cch* and *gun5* mutants the observed reduction in chlorophyll was attributed to defective Mg-PP IX synthesis (Mochizuki et al., 2001). Likewise, *in vitro* characterization of the recombinant *Synechocystis* 6803 ChlH proteins showed both mutations abolished Mg-chelatase activity; however, Mg-chelatase activity was partially restored with the addition of the Gun4 protein (Davison and Hunter, 2011). The crystal structure of *Synechocystis* ChlH reveals that Pro595 and Ala942 are in the central cage-like structure of the protein that houses the catalytic site (Chen et al., 2015). However, while the Gly195 to Glu substitution in our *Synechocystis* 6803 ChlH-G195E strains also resulted in reduced chlorophyll *a*, the Gly195 residue is not in the catalytic cage-like domain. Therefore it is not clear whether the reduction in chlorophyll *a* in our mutants is caused by impaired catalytic activity or by an indirect mechanism. The proximity of Gly195 to the inter-dimeric salt bridge formed by Glu199 and Lys1018 might mean the Gly195 to Glu substitution could affect the formation and stability of the ChlH dimer; however, the physiological relevance of the ChlH dimer in chlorophyll *a* biosynthesis is unknown. In addition, the residues involved in salt bridge formation were not conserved in all cyanobacteria.

### Combining the ChlH-Gly195 to Glu Mutation in a ΔYcf48 Strain Abolishes Photoautotrophic Growth

During PS II biogenesis and repair Ycf48 is transiently associated with pD1-containing pre-complexes prior to D1 processing and subsequent formation of the RC47 assembly intermediate (Komenda et al., 2007, 2008, 2012). In this study when the absence of Ycf48 was combined with the ChlH Gly195 to Glu mutation, the resultant double mutant showed limited photoautotrophic growth, a decreased level of PS II activity and a decrease in levels of assembled PS II dimers, monomers and the RC47 assembly intermediate compared with the GT-O1:G195G:ΔYcf48 strain (**Figures 4–6**). We also observed that PS I assembly was destabilized in our GT-O1:G195E:ΔYcf48 strain, perhaps as an indirect consequence of perturbing nascent PS II assembly and repair complexes. A similar effect on PS I assembly in an *A. thaliana* mutant lacking HCF136 – the homolog of Ycf48 – has also been reported (Plücken et al., 2002).

Given the close relationship between chlorophyll biosynthesis and the assembly and repair of PS II (Vavilin et al., 2007; Yao et al., 2012; Chidgey et al., 2014; Knoppová et al., 2014) and PS I (Kopečná et al., 2013; Hollingshead et al., 2016) manipulating the supply of chlorophyll may reveal novel roles for assembly factors of both PS II and PS I as seen in our GT-O1:G195E:ΔYcf48 strain.

## Author Contributions

TC performed the experiments and wrote the first draft of the manuscript. TS and JE-R designed the experiments, wrote, and edited the manuscript.

## Conflict of Interest Statement

The authors declare that the research was conducted in the absence of any commercial or financial relationships that could be construed as a potential conflict of interest.
