# Supplementary material for: Mutation of Gly195 of the ChlH Subunit of Mg-chelatase Reduces Chlorophyll and Further Disrupts PS II Assembly in a Ycf48-Deficient Strain of Synechocystis sp. PCC 6803
Source: Front Plant Sci. 2016 Jul 20;7:1060. doi: 10.3389/fpls.2016.01060 (PMC4951491; doi:10.3389/fpls.2016.01060)
Supplement: Supplementary file 1 [file Data_Sheet_1.PDF]

SUPPLEMENTARY INFORMATION

Mutation of Gly195 of the ChlH subunit of Mg-chelatase reduces chlorophyll and further disrupts PS II assembly in a Ycf48-deficient strain of *Synechocystis* sp. PCC 6803

Tim S. Crawford[1,2], Julian J. Eaton-Rye[1], Tina C. Summerfield[2*]

[1]Department of Biochemistry, University of Otago, Dunedin 9054, New Zealand

[2]Department of Botany, University of Otago, Dunedin 9054, New Zealand

***Correspondence:**
Tina Summerfield

tina.summerfield@otago.ac.nz

Construction of ChlH mutant strains

The ChlH mutant strains used in this work were constructed using an overlap-extension PCR-based method (Bryskin & Matsumura 2010). The oligonucleotides used for overlap extension PCR are listed in Table S1; a subset of these were designed to amplify the sequence of interest and introduce a ~10 bp sequence from the adjacent component in the final construct.

A DNA fragment was generated using overlap-extension PCR, in which most of the *slr0168* gene was replaced by a spectinomycin-resistance cassette (Fig. S1A, B). This fragment was generated using the three PCR products outlined below. A 1014 bp amplicon that included 911 bp of *slr0338* and the first 18 bp of *slr0168*, amplified using the primers dslr0168-lff and dslr0168-lfr. The reverse primer dslr0168-lfr included a 10 bp overlap with the spectinomycin-resistance cassette. A 1487 bp PCR product containing a 1466 bp spectinomycin-resistance cassette, which contained the *aadA* gene, was amplified using the primers dslr0168-specf and dslr0168-specr. These primers overlap with sequences in the *Synechocystis* sp. PCC 6803 (hereafter *Synechocystis* 6803) neutral site, these overlaps regions were 11 bp and 10 bp for the dslr0168-specf and dslr0168-specr primers, respectively. In addition, the primer dslr0168-specf was designed to introduce a HindIII site at 15 bp from the 5' of the spectinomycin-resistance cassette. The third PCR product was 1000 bp and contained the *ssl0138* and *slr0169* genes along with the 3' 54 bp of *slr0168*, amplified using the primers dslr0168-rff and dslr0168-rfr. The primer dslr0168-rff contained an 11 bp region of the spectinomycin-resistance cassette.

These amplicons were generated by PCR amplification using *Synechocystis* 6803 wild-type gDNA or template DNA containing the spectinomycin-resistance cassette as appropriate. Separate reactions were performed for each of the three primer sets and the products were purified and used as templates for the overlap extension PCR with primers dslr0168-lff2 and dslr0168-rfr2. The resulting 2957 bp amplicon was purified and ligated into pGEM-T Easy resulting in the Δ*slr0168*:specR plasmid. This plasmid was used to transform wild-type *Synechocystis* 6803 (GT-O1) to create the Δ*slr0168* neutral site control strain (Fig. S1B).

Generation of ChlH mutants was performed in two steps. In the first step, the *slr1055* (*chlH*) gene was amplified from the GT-O1 and GT-O2 strains (Morris et al., 2014), using the primers ChlH_Hindf and ChlH_Hindr, which were designed to introduce HindIII restriction sites at the ends of the amplicons. These primers generated 4364 bp amplicons containing the *chlH* gene with 212 bp and 156 bp upstream and downstream of *chlH*, respectively. These amplicons and the Δ*slr0168*:specR plasmid, were digested with HindIII and ligated to produce the ChlH-G195G:specR and ChlH-G195E:specR plasmids. These plasmids were transformed into *Synechocystis* 6803 strains (GT-O1 or GT-O2) to introduce either the unmodified *chlH* (in the GT-O1:G195G and GT-O2:E195G strains) or the G195E mutant *chlH* gene (in the GT-O1:G195E and GT-O2:E195E strains) into the putative neutral site (Fig. 2A).

Subsequently, a Δ*chlH* plasmid was constructed using overlap-extension PCR, in which a chloramphenicol-resistance cassette was inserted at the site of the native *chlH* (*slr1055*) gene, resulting in deletion of the *chlH* gene, except the 5' three bases and additionally deleting 10 bp of the 3' of *chlH* gene. The three amplicons used for overlap extension PCR are outlined below (Fig. S1C, D). The left flank was a 998 bp product, containing 501 bp of the *sll1009* gene and the 487 bp upstream of *slr1055*, that had been amplified using the primers dchlHc-lff and dchlHc-lfr. In addition, the dchlHc-lfr contained a 10 bp region of overlap with the chloramphenicol-

resistance cassette. A 1229 bp region of a chloramphenicol-resistance cassette containing the *cat* gene, was amplified using the primers dchlHc-chlrf and dchlHc-chlrr. These primers contained 10 bp regions of overlap with the left and right flank amplicons, respectively. The right flank was a 977 bp fragment containing sequences downstream of *slr1055* including *slr1056*, this was amplified using the primers dchlHc-rff and dchlHc-rfr. The primer dchlHc-rff contained a 10 bp overlap with the chloramphenicol-resistance cassette sequence in primer dchlHc-chlr. These three amplicons were generated using *Synechocystis* 6803 gDNA or DNA encoding the chloramphenicol-resistance cassette as templates. The PCR products were purified and used as templates for the overlap extension PCR with nested primers dchlHc-lff2 and dchlHc-rfr2, yielding a 2933 bp PCR product. This amplicon was purified and ligated into pGEM-T Easy to produce the Δ*chlH* plasmid. This plasmid was transformed into the GT-O1:G195G, GT-O1:G195E, GT-O2:E195G and GT-O2:E195E strains which each contained a second copy of the *chlH* gene in the putative neutral site, to produce strains which each contained only one copy of *chlH*, either the GT-O1 (G195G) or GT-O2 (G195E) variant.

Construction of the Δ*ycf48* (Δ*slr2034*) plasmid has been described previously (Jackson et al., 2014); this plasmid was transformed into the GT-O1:G195G and GT-O1:G195E strains to produce GT-O1:G195G:ΔYcf48 and GT-O1:G195E:ΔYcf48 strains, respectively.

SUPPLEMENTAL TABLE SI

Oligonucleotides used for construction and verification of the ChlH mutant strains.

Name	Sequence (5′-3′)
Δ*slr0168* neutral site plasmid construction	
dslr0168-lff	TAGACACCGCCAGTAAGTATCG
dslr0168-lfr	GAGATCGGAAGAGGTGTATTGAATAGTCATAG
dslr0168-specf	TTCAATACACCTCTTCCGATCTCCTGAAGCTTGGGC (HindIII)
dslr0168-specr	CTCCTCCGCCTACTTCGCCAACTATTGC
dslr0168-rff	TTGGCGAAGTAGGCGGAGGAGACCTAGATTTCG
dslr0168-rfr	TTACGCTTCCATCTCACTA
dslr0168-lff2	TCAAAACGGAAGAGTTATTGGC
dslr0168-rfr2	AACTACCGATGGAACTAACCTGC
Δ*chlH* plasmid construction	
dchlHc-lff	AGTCCCCGTCAGGGTTAG
dchlHc-lfr	TGAACTGCAGATAAGTGGTGTTCTCCTAAA
dchlHc-chlrf	CACCACTTATGGGTTCCGCGCACATTTC
dchlHc-chlrr	TTAGTTCAGGCGTCAATTATTACCTCCACG
dchlHc-rff	ATAATTGACGCCTGAACTAAGATATAGGGG
dchlHc-rfr	TAAATTTGCTAGTGCTTTAT
dchlHc-lff2	TCACTCAGAAACAAATATGC
dchlHc-rfr2	GGATTCATCATCAAATAGTAATGC
ChlH_Hindf	TACCATTAGTTTTAAGCTTAGCAC (HindIII)
ChlH_Hindr	TGTTCTTTAAGTTAAGCTTTG (HindIII)
slr2034f	CGGTGAACCAATGATGAATCC
slr2034r	AACCATGATATTGTGTGGCGG

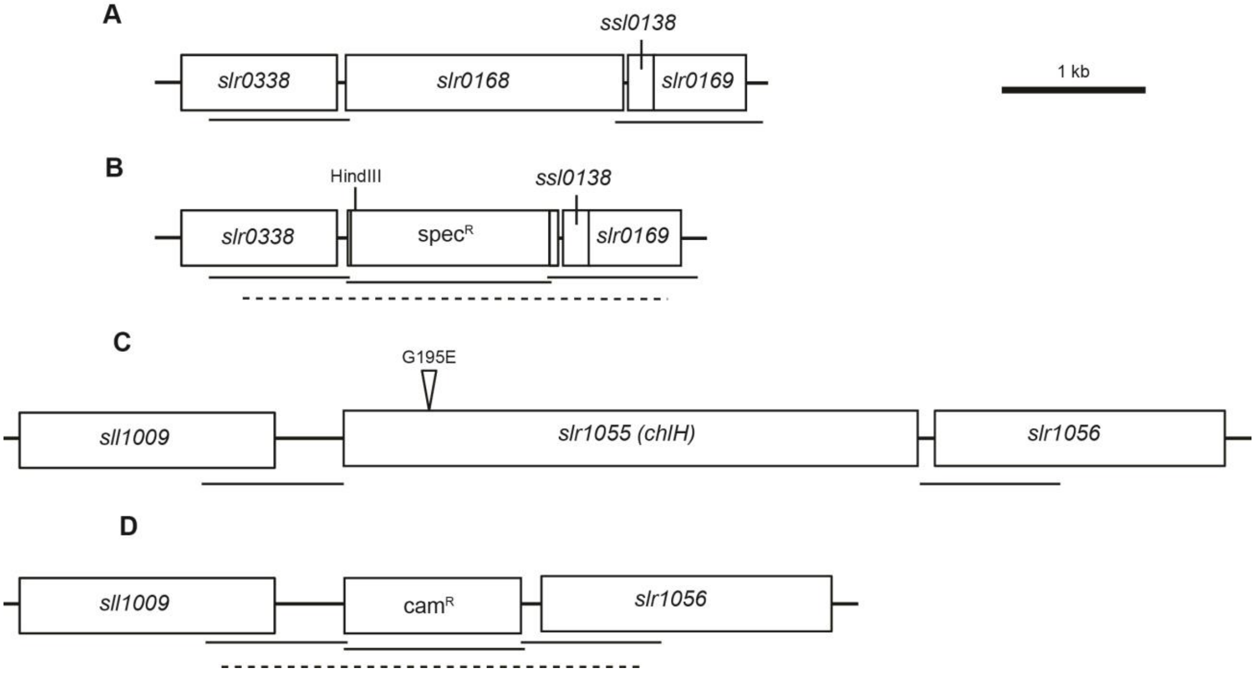

Figure S1. Construction of the mutant strains of *Synechocystis* sp. PCC 6803 using overlap extension PCR. A) Genomic structure of the *slr0168* neutral site region in the wild-type strain. Black lines under *slr0338* and *ssl0138/slr0169* indicate the PCR products generated for the overlap extension PCR production of the *slr0168* neutral site control strain. B) Genomic structure of the *slr0168* neutral site in the Δ*slr0168* neutral site control strain. The majority of the *slr0168* ORF has been replaced with a spectinomycin-resistance cassette containing a HindIII restriction site. The black lines under the genes indicate the three PCR products that were used to make the overlap extension product, this amplicon is presented as a dashed line. C) The *slr1055* (*chlH*) region of the *Synechocystis* 6803 chromosome. The position of the Gly195 to Glu substitution present in the GT-O2 wild-type strain is indicated by a triangle. Black lines under the genes indicate the two PCR products generated for the production of the *chlH* deletion construct. D) Genomic structure of the *slr1055* (*chlH*) region in the *chlH* mutant strains. The native *chlH* ORF was replaced with a chloramphenicol-resistance cassette using a construct generated from the three PCR products shown as black lines under the genes. These PCR products were used to produce the overlap extension PCR product shown as a dashed line. This construct was used to form the GT-O1:G195G, GT-O1:G195E, GT-O2:E195G and GT-O2:E195E strains, which contain only one copy of *chlH* introduced into the neutral site, from their precursors.

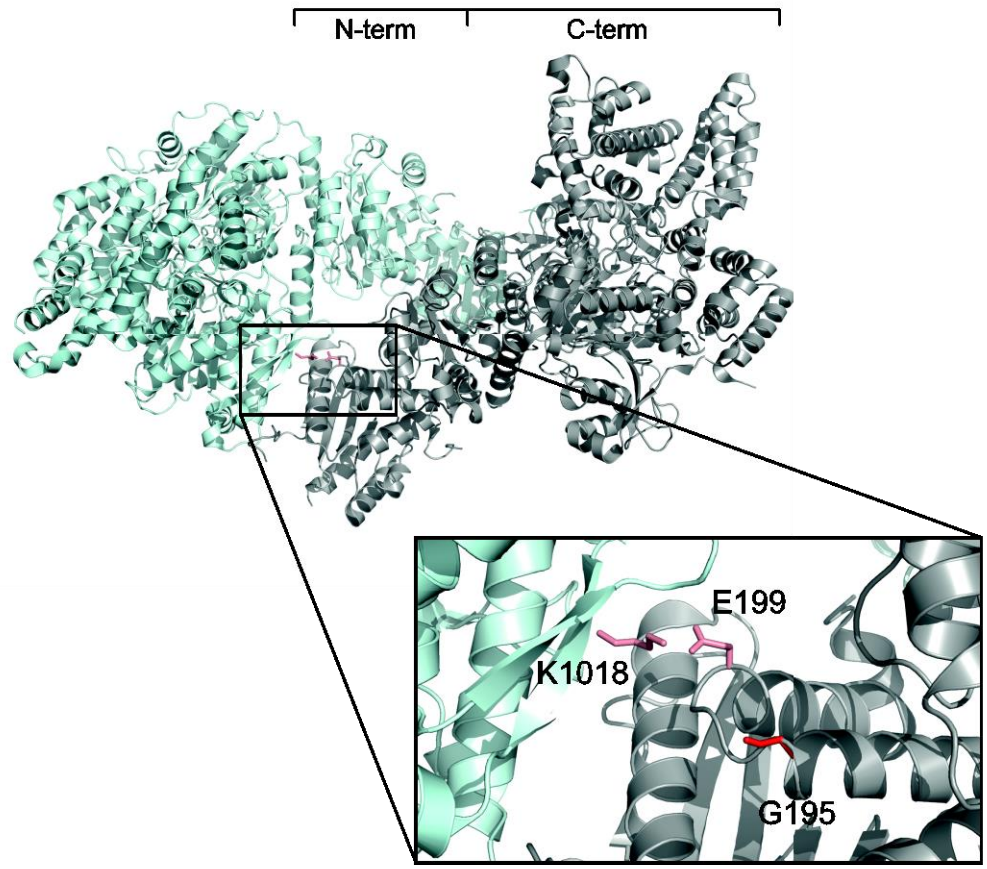

Figure S2. Position of the Gly195 residue in the crystal structure of the ChlH subunit of the Mg-chelatase enzyme from *Synechocystis* sp. PCC 6803. Two monomers of ChlH are shown as cyan and grey, with the N-terminal 'head/neck' (domains I and II, respectively) and the C-terminal 'cage-like' structure (domains II-VI) of the grey monomer indicated. The monomer-monomer interface is shown in more detail in the inset; the positions of the Lys1018 residue of the cyan monomer and the Glu199 residue of the grey monomer are shown as pink sticks, and the Gly195 residue of the grey monomer is shown as red sticks. PDB: 4ZHJ; Chen et al. (2015).

References

Bryskin, A. V., and Matsumura, I. (2010). Overlap extension PCR cloning: a simple and reliable way to create recombinant plasmids. *Biotechniques* 48, 463–465. doi:10.1016/j.biotechadv.2011.08.021.

Chen, X., Pu, H., Fang, Y., Wang, X., Zhao, S., Lin, Y., et al. (2015). Crystal structure of the catalytic subunit of magnesium chelatase. *Nat. Plants* 1, 15125–15129. doi:10.1038/nplants.2015.125.

Morris, J. N., Crawford, T. S., Jeffs, A., Stockwell, P. A., Eaton-Rye, J. J., and Summerfield, T. C. (2014). Whole genome re-sequencing of two "wild-type" strains of the model cyanobacterium *Synechocystis* sp. PCC 6803. *New Zeal. J. Bot.* 52, 36–47. doi:10.1080/0028825X.2013.846267.
